# Ageing and Keeping Pace with Technology: A Grounded Theory Study on Blind Adults’ Experiences of Adapting to New Technologies

**DOI:** 10.3390/ijerph20031876

**Published:** 2023-01-19

**Authors:** Alina Betlej, Jan Gondek, Natalia Gondek

**Affiliations:** 1 Centre of Sociological Research on the Economy and the Internet, The Department of Economic and Digital Sociology, The John Paul II Catholic University of Lublin, Al. Racławickie 14, 20-950 Lublin, Poland; 2 The Department of Economic and Digital Sociology, The John Paul II Catholic University of Lublin, Al. Racławickie 14, 20-950 Lublin, Poland; 3 The Institute of Philosophy, The John Paul II Catholic University of Lublin, Al. Racławickie 14, 20-950 Lublin, Poland

**Keywords:** ageing, keeping pace with technology, technologies for blind adults, digital development, digital exclusion, age-friendly technologies

## Abstract

This study investigated blind adults’ experiences of adapting to digital technologies. The authors’ focus was on how they have been experiencing changes implied by digital transformation, which provided the starting point for assessing their prospects and/or limitations for self-development through new technologies in the future. The second point concerned designing technologies for blind adults by adding questions about their specific needs and expectations for digital device designers. To develop these main issues, we planned a qualitative grounded theory study in which 16 blind adults were individually interviewed. It aimed to provide rich descriptions of a selected phenomenon. The research analysis was conducted by using the sociological and philosophical methods, which provided insights into the social assessment of digital development for ageing by blind adults. The data analysis revealed five distinct categories that captured these experiences and expectations: (1) wishing to learn—“Digital technology gives me privacy and independence”; (2) having to learn—“If you do not have new digital devices or do not know how to use them, you end up on the margins of society”; (3) being socially excluded—“Designers of new technologies do not think about blind adults”; (4) fearing to stop understanding—“Technological development is too rapid, it is difficult to be on time while ageing”; and (5) waiting for the changes—“I hope my situation will change in the future”. Together, these five categories form the basis of the core category “Ageing and keeping pace with technology”, which encapsulates the initial adaptation experiences of the interviewees to the technological development. The findings indicate that the blind adults experienced digital devices as tools for improving their well-being, but they also saw them as posing the threat of being socially excluded because of new technology designs and accessibility barriers.

## 1. Introduction

Blindness and visual disability are one of the most serious problems that may affect a person worldwide [1,2]. Visual impairment is considered to be curable [3,4]. In the case of blindness, besides the great efforts spent in medicine, no solutions have been found so far. In contrast, the great efforts spent in developing digital technologies to support them seem to give a promise for improving their well-being and possibilities of self-development in modern technology-driven societies [5,6,7]. Technological advancement also brings the peril of social exclusion of individuals who have no access to new solutions and/or have a low level of digital skills [8].

Furthermore, the social situation of blind people varies greatly from country to country [9]. In many societies, there are still stereotypes about blind people, who are perceived as being incapable of using a computer, laptop, smartphone, and other similar digital devices. The social situation of blind people is often difficult [10]. They have problems finding jobs that are compatible with their education. It also still happens that they rely on the help of third parties, especially family [11]. The digital competence of blind people is rated as low [12]. They are rarely seen as independent individuals, fully prepared for independent living and gainful employment. In Poland, blind people are not a frequent subject of research referring to issues of technological development.

There are limited empirical qualitative studies devoted to these issues. In Poland, no research based on the in-depth individual interviews or focus groups has been conducted so far that would address the problem of experiencing technological development by blind adults. In this grounded theory study, the authors used more general questions about blind adults’ experiences in using new digital devices, assessment of their digital competences, descriptions of their specific needs for new solutions in the context of ageing, and their feelings about their prospects for future self-development. Blind adults are often perceived as digitally excluded in Poland, despite the low number of research studies in this topic [13]. Therefore, it was interesting to conduct this qualitative study and to discuss the potentials for change of the blind adults’ individual and social situation in contemporary technologically advanced societies. The most interesting question is about the possibilities of cooperation between blind adults and designers of new technologies.

The paper is structured as follows: Section 2 outlines the literature review of the social situation of blind adults in contemporary technologically advanced societies. Section 3 provides an overview of the methodology, while Section 4 highlights the research findings. The discussion in Section 5 provides answers the research question in light of previous findings and literature review. Section 6 discusses the research limitations and suggests directions for future studies. Finally, Section 7 offers the research conclusions.

## 2. Literature Review

The professional and social activity of blind people is conditioned by a complex set of internal and external factors. In the literature, we find many examples of specific problems faced by people with disabilities [14,15,16]. According to numerous research studies, the situation of blind people is usually difficult [17,18,19]. They have problems finding work in general, and particularly not only in line with their education [20]. Unfortunately, it is still the category of people that they rely on the help of third parties, especially family. Negative stereotypes about blind people still exist perpetuated in many societies [21]. They are perceived as incapable of using a computer, laptop, smartphone, or other similar digital devices [22]. Blind people are thus at risk of social exclusion. Stereotypical perceptions of the professional roles of blind people seem to be crucial in the process of social assessment of their individual, social, and professional potential [23]. The abovementioned functioning ways of thinking about people with disabilities are just some of the examples of the barriers to their social activity.

Previous research results have shown that the immediate social environment plays a major role in the education of blind people and in building their career paths [24,25]. The knowledge and specialist competence of parents, siblings, or other caregivers was not without influence on the future choices of people with disabilities. It should be emphasized that blind people are one of the categories of people with disabilities most dependent on third-party assistance.

Other barriers hindering the social inclusion of blind people include the lack of satisfactory institutional solutions, architectural barriers, and the maladjustment of the education system to the changing conditions of the labor market, especially with regard to the improvement of professional competences [26,27,28]. Much attention in the literature has been given to adapting of urban spaces to the needs of blind people [19,29,30,31,32]. The inadequate infrastructure of public space hinders their independent functioning. Space is described as a factor of social exclusion [33]. In order to counteract this, designers and space managers need to eliminate and reduce barriers in the built environment, including urban, architectural, and transport barriers, but also awareness and organizational barriers.

In an effort to meet the needs of visually impaired people and provide them with better living and working conditions, a number of projects have been set up around the world in recent years [34]. From a general perspective, these can be divided into two main streams. The first addresses the issue of guiding/navigating blind people to avoid potential obstacles [35]. The second focuses on recognizing the nature of nearby moving/static obstacles [36]. More and more of these types of solutions are being implemented around the world and are becoming elements of modern smart cities. Research in these streams has paid much attention to the importance of creating welcoming spaces through the use of new technologies, such as artificial intelligence, virtual reality technologies, ICT, robots, etc. [37]. Their results direct our attention to the question of the need to know the needs of blind and visually impaired people for such solutions [38,39]. The theoretical framework for investigating the needs and challenges faced by blind people entering the labor market is most often formed by the concepts of social inclusion and social exclusion, and less often by the network society or the digital society [40,41]. The social policy, economics, functionality, and aesthetics of spatial solutions are the most frequent topics of scientific works that address the problems of blind people [42]. The issue of harnessing the potential of digital services to support the social integration of blind people is now an important issue of international legislation, which aims to create a framework of technical standards for facilities and devices designed for them [43].

The development of new digital technologies seems to create new opportunities for the social integration of blind people by developing new forms and methods of education and supporting their active participation in the open labor market [44]. Moreover, many of today’s social changes are the result of technological developments [45]. We live in increasingly digitized societies in which digital technologies are becoming an indispensable part of human activity [46]. It can be argued that digital technologies are becoming an invisible agent of social interaction [47,48]. More and more attention is therefore being paid to the ambivalent effects of this development [49]. The problem of the relationship between technical and social development is nowadays the subject of interdisciplinary discussions, the context of which is social transformation of a previously unknown scale. It is an internally differentiated problem [50].

The literature review context reveals interesting research gaps. There is still a lack of knowledge about the most important factors influencing blind adults’ attitudes toward technological development. Poland is an example of an ageing society [51]. Blind people are very often considered to be the social category of being at risk of digital exclusion and social exclusion understood in a broader sense [52]. Digital devices are better adopted by young people [53]. Therefore, it is interesting to conduct a qualitative study to see how blind adults perceive designing age-friendly technologies for them in different life stages. In Poland, no solution has yet been developed that would enable blind people to improve their competences and acquire professional qualifications remotely via a professional e-learning platform that meets all accessibility standards and is fully supported by reading programs. An important task is to find out the opinion of people who face various problems in their daily life related to accessibility or lack of access to certain technical solutions. A second important issue, which has not yet been sufficiently addressed in the Polish literature, is the assessment of the level of digital competence of blind people. The third problem is an attempt to answer the question about the real needs of blind people’s participation in the process of designing digital tools friendly to them. The authors aim to fill these knowledge gaps by providing a Polish blind adults’ perspective on experiencing technological development and needs of participation in designing age-friendly technologies for the blinds. To proceed with the research, however, we did not create any pre-conceptualisations, as we adopt the assumptions of the grounded theory. The detailed methodology and research procedure is described in the following sections of the paper.

## 3. Materials and Methods

### 3.1. The Design/Methodology/Approach

The authors used a grounded theory approach developed by Strauss and Corbin to explore the blind adults’ perception of digital development in order to understand it from the social context of ageing and keeping pace with technology [54,55]. The second point concerned their specific technological needs and expectations for digital devices designers. In classical sociology, the gap between the ‘colloquiality’ of life and the ‘scientificness’ of the theory of that life is apparent. Typically, therefore, the research data are reflected through the prism of the researcher’s chosen theories. There are many different theoretical approaches in classical sociology. However, the differences between them are considered to be secondary, as the methodological procedure mentioned above remains valid at all times. A scholar who has made a significant difference in the sociological reflections to the concept of theory is Anselm Strauss [56]. He had a completely new understanding of the concept of grounded theory that was hitherto unheard of in sociology. Glaser and Strauss’ main goal of their deliberations was to orient the grounded theory research methodology around the ‘rendition of reality’ [57]. This is because social reality is inscribed with a certain abstract theoretical schema, which emerges gradually during the course of the research process. Grounded theory is based on the assumption that social reality is best understood by the actors involved in it. Consequently, the researcher of a given social reality rejects the traditional functionalist approach in which they analyze it using a pre-developed theoretical model. This is because they recognize that such a procedure would only lead to the self-confirmation of a given theory. The researcher sets out into the field to conduct research without using pre-conceptualized theories. As they collect research material in subsequent interactions, the theory emerges from the research itself. The theory that thus emerges can be referred to as a ‘factual theory’, evolving as the ‘research facts’ are collected, developed and analyzed. Moreover, it can also lead to the discovery and elaboration of gaps in the formal theory. It can be said that the concept of the grounded theory bridges the gap between the immediacy of the factual theory and the abstract generality of the formal theory.

A grounded theory approach that is consistent with the work of Strauss and Corbin was chosen as it facilitated the development of a new perspective on the experiences of blind adults adapting to new technologies. Grounded theory is recommended when one is investigating social problems or situations to which people must adapt. Grounded theory is an interesting methodology that can be used to understand the actions and processes through the transitions. Crucial to this methodological procedure is the process of data collection, and later, coding and categorisation. This will be described in the following sections of this paper.

These assumptions led the researchers to develop a qualitative interview scenario. An open-ended interview schedule was designed to stimulate discussion about the individuals’ perceptions, thoughts and feelings about their early experiences in adapting to new technologies. It provided rich descriptions of a selected phenomenon, such as the ways of perceiving ageing, digital development, opportunities, and challenges of technological development for blind adults and gave insights into assessment of the future prospects for self-development for blind people with digital technologies. The researchers assumed that creating particular conceptions of social problems is related to the research type and the specificity of the data. This scientific avenue is based on philosophical and sociological considerations which imply the path to understanding the individual and social backgrounds, such as adaptability, social imagination, experiencing digital change in the context of ageing and keeping pace with technology by blind adults who are at risk of social exclusion [58]. The authors used the methods of criticism of writing and analytical and synthetic methods to interpret the theoretical and empirical studies in the second section. They developed a constructive approach to go beyond the paradigm of cause-and-effect thinking [59,60]. Before conducting the interviews, the authors developed a list of information they were looking for, which was important to them in terms of the issue under investigation. They assumed that they would be open to new, emerging circumstances, as well as information, during the interviews. The researchers also assumed that they could change the order of the questions during the interviews, as well as their form and content, to suit the respondents.

To this aim, the authors articulated the following leading questions, which were developed into more specific questions during the interview:

1. How do blind adults assess and describe their digital competences?

What digital technologies/devices do you use on a daily basis? For what purposes do you use them? How do you use them? What makes it difficult for you to use these devices? When did you start using these devices? Who helps you to acquire new digital skills? How do you assess the digital skills of visually impaired/blind people in Poland? Why do you rate them this way? Considering your daily experience with visually impaired/blind people, which people are best at using new technologies? How do you feel, and what influences this? Education; age; access to new technologies; people’s individual attitudes, e.g., openness to new challenges; their interests, etc.

2. Which specific technologies for blind people are best appreciated by respondents, taking into account their individual experiences or knowledge? If you had to list the 5 most important technologies for blind adults, what examples would you give? Why are these technologies so important? Are these technologies widely available to blind adults in Poland? If you could choose any technology (e.g., gadget, software, etc.) intended for blind adults which is available on the market, but which you currently do not have access to for various reasons (e.g., price), what would it be? Do you keep track of information on technological innovations for blind adults?
3. How do we define age-friendly technologies for blind people?

When you hear the phrase “technology for older people”, what do you think of? What associations does it make for you? Imagine yourself in 40 years’ time, you are already an older person, and you are no longer working professionally. What technologies, technical solutions, will you need then? Exactly what functions should this device have (e.g., robot, gadget, phone, app, etc.), and what could it be used for? How do you feel? Do the needs of blind people to use new technologies change as they get older? How do they change? What do younger people expect/need, and what do older people need?

4. How do blind adults experience the design of digital devices?

Are designers of new technologies creating solutions that meet the needs of blind people? Have you ever been involved in designing technical solutions for the blind? Would you like to take part in such an experiment/project, where you could actively participate in designing new technologies for blind people? What could your role be? What are your feelings about the ethical risks of designing/developing new technologies for visually impaired people?

### 3.2. Participants/Sampling

In order to select the participants, it applied the purposive sampling strategy of maximum variation, which was initiated by “identifying differential characteristics or criteria for constructing the sample” [60]. In this study, the considered characteristics were gender, age, and being blind from birth. Researchers recruited blind adults aged from 26 to 40+ in order to interview people who have experienced the digital turn toward touchscreens and mobile devices (see Table 1). The assumption was made that older blind people in Poland could not have had opportunities to learn these new solutions and would have difficulties in assessing the future of digital technologies for those at risk of digital exclusion. The snowball sampling technique was applied to target the interviewees. The sample consisted of 16 respondents, men and women living in the Lublin Region (Poland), mainly towns (see Table 1).

Since the framework of the analysis is a grounded theory approach, the second sampling method used by researchers was theoretical sampling/data saturation. The logic behind this sampling strategy is that, in the coding process, sampling proceeds on the basis of concepts derived from evolving theory and on the basis of the concept of “making comparisons”, the aim of which is to go to places, people, or events that will maximize the possibilities of discovering variations among concepts and identifying categories in terms of their properties and dimensions and will be discontinued when there are no more new things to be known and theoretical saturation is reached. According to these two sampling strategies, the researcher continues sampling, data collection, and data analysis until no new insights are generated through the ongoing sampling process and the implementation of additional interviews would not significantly contribute to solving the research problem.

Participation in the interviews was voluntary. The respondents were informed about the purpose of the study, the research data management procedure and about anonymity. The interviewees were also assured of their right to refuse to participate in or to withdraw from the study at any stage. They all have agreed to participate in the interviews. The interviewees are identified by codes in the paper (see Table 1). The study protocol was approved by the Research Ethics Committee of the John Paul II Catholic University of Lublin.

### 3.3. Data Collection

Data were collected through in-depth open-ended interviews. The aim of the researchers was to establish a dialogue with the participants about their experiences of using digital technologies for blind people and to assess the potentials and limitations that digital developments bring for this category of people. The interview scenario allowed for a running interpretation of the descriptions provided by the participants and some conceptual reconciliation. The in-depth semi-structured interview was the data-collection tool. A feature of the in-depth strategy is that the answers given continually inform the unfolding conversation [61]. In the case of semi-structured interviews, the same thematic issues were addressed in each interview. However, each time, participants were free to add new issues related to the interview topic. The interview framework was not completely predetermined and standardized. The interviewer had opportunities to arrange conditions that were conducive to fully engaging in the interviews and telling their stories and experiences. The researchers assumed that following the grounded theory approach it is the subjective lenses of narrators to be central to describing the experience of a research phenomenon.

The interviews were conducted with 16 blind adults between March and July 2022. The interviews were held at places chosen by participants and ranged from 45 to 60 min. The interview process was stopped when theoretical saturation was reached. The criterion for saturation was that the researchers did not come to new data or concepts after interviewing the 16 participants. The interviews were audio-recorded and transcribed verbatim following the clean, verbatim transcriptions rules. In two cases where the participants did not consent to be recorded, the interviews were transcribed manually by the researcher during the interview. The transcription process took between 5 and 6 h for each interview so that the transcriptions could then be analyzed. Each of the transcriptions were conducted by one of the authors who interviewed this participant. To ensure that the transcriptions were as accurate as possible, all authors listened to the interviews to compare the transcriptions with the audiotapes of the interviews and modified any grammatical and spelling errors. The transcribed data were coded by using an inductive approach to identify concepts/themes representing blind adults’ experiences of using digital technologies. A second method of rigor in identifying the concepts analyzed was ensured by double-checking each code. Reliability in coding was ensured by performing a cross-coding analysis of the coded data. Individual coding was carried out by the author interviewing that participant and, after the procedure, Authors 2 and 3 checked and validated the developed coding system. The Krippendorff alpha coefficient (α) was used to quantify the degree of agreement between coders. Values of the coefficient could range from 0 to 1, where 0 represents perfect disagreement and 1 represents perfect agreement. Krippendorff suggests requiring α ≥ 0.8. Where tentative conclusions are still acceptable, α ≥ 0.667 is “the lowest conceivable limit” (Krippendorff 2018). In the study, Krippendorff’s alpha coefficient (α) was 0.85, and the concordance between the two coding sets was interpreted by the authors as strong.

Following the second sampling method, which was data saturation, during the current analysis of the interviews with blind adults, by the fifteenth interview, about 90% of all codes were identified. This situation is explained as data saturation, i.e., a situation in which the implementation of additional interviews would not contribute significantly to solving the research problem. It was therefore assumed that the sample size was not a limitation of the research goals.

### 3.4. Data Analysis

The gathered data were very subjective and rich. Thus, the analysis entailed reading a large number of transcripts and looking for similarities or differences, finding themes, and developing categories. The transcriptions of the interviews were read several times, following a narrative method that allows for reflexive understanding of the participants’ experiences. The collected data were evaluated by using the chosen coding approach interpreted as conceptual abstraction involving the assignment of general concepts (codes) to individual incidents occurring in the data. The data analysis was framed within a qualitative grounded theory methodology as a dynamic, intuitive, and creative process of inductive reasoning, thinking, and theorizing. The study is based on three kinds of coding procedures, namely open, axial, and selective coding, to analyze the data collected through the interviews. Open coding was focused on the conceptualization and categorization of phenomena through an intensive analysis of the data. Axial coding was implemented to investigate the relationships between concepts and categories that have been developed in the open coding process. The last phase of selective coding was adopted in order to integrate the different categories that have been developed, elaborated, validated, and mutually related during axial coding into one cohesive theory. As a result of the coding process, the statements of the interview participants were categorized accordingly. The next stage of the research procedure was to select the respondents’ statements that were representative of the given thematic categories in the MAXQDA software. These statements are quoted in the paper.

**Figure 1:**
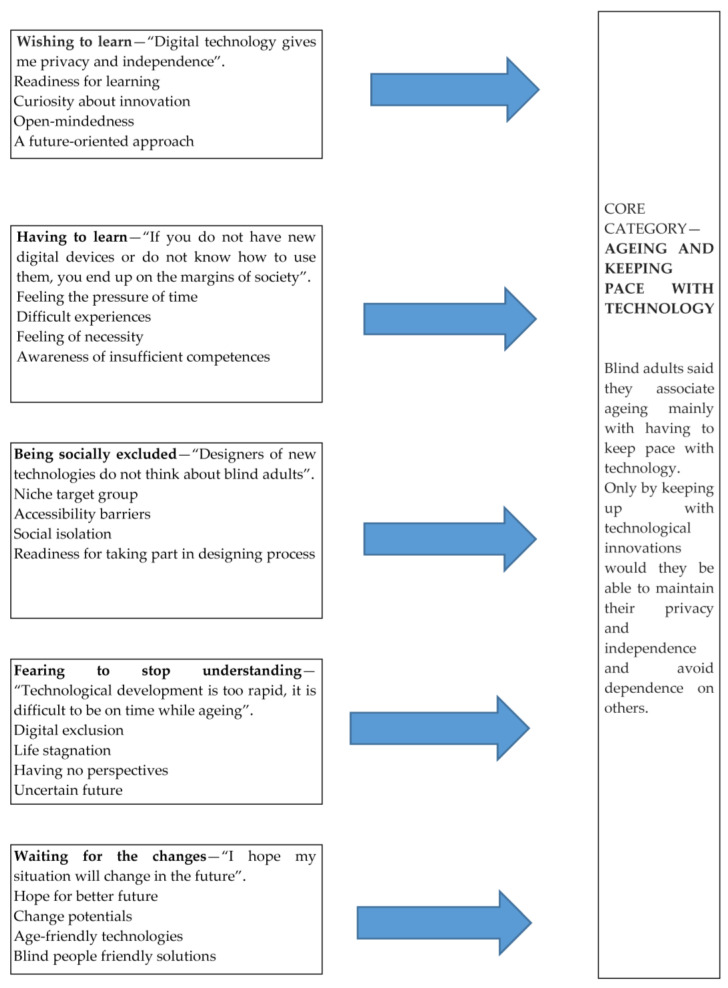
Diagram illustrating the relationship of major categories to each other and to the core category.

To reach this goal, the researcher focused on the exploration of values, meanings, beliefs, thoughts, experiences, and feelings specific to the phenomenon under study. The transcripts were systematically searched and arranged to increase the understanding of how the participants perceive designing robots for older people. The process of data analyzing predominantly involved coding or categorizing the data and was the most important stage in the process. The researcher reduced the raw information and then identified meaningful patterns and hidden meanings from the data to build a logical chain of evidence. The data were subsequently assigned into categories of identified themes or topics compiled in the study (see Figure 1). The process of conducting interviews and the analysis of their transcripts were carried out in Polish language.

## 4. Results

In total, the coding process yielded over 180 concepts, of which 150 relevant concepts were eventually used, creating a total of 5 categories (see Figure 1). The number of concepts in each category ranged from 13 to 25. These categories include (1) wishing to learn—“Digital technology gives me privacy and independence”; (2) having to learn—“If you do not have new digital devices or do not know how to use them, you end up on the margins of society”; (3) being socially excluded—“Designers of new technologies do not think about blind adults”; (4) fearing to stop understanding—“Technological development is too rapid, it is difficult to be on time while ageing”; and (5) waiting for the changes—“I hope my situation will change in the future”. Together, these five categories form the basis of the core category “Ageing and keeping pace with technology”, which encapsulates the initial adaptation experiences of the interviewees to the technological development. The categories that emerged are presented in the following section, along with illustrative quotes of the interviewees.

### 4.1. Wishing to Learn—“Digital Technology Gives Me Privacy and Independence”

Many interviewees had positive attitudes toward learning new digital skills and experimenting with new technologies. They declared wishing to learn new things, competences, and how to handle new digital equipment. These attitudes were supported by encouragement from their families, friends, and caregivers.

“I wish to have the opportunity of life-long learning in all areas of my life. It is my dream to be on time, to be aware of what is going on around me. First of all, I want to be independent. My parents have always told it. […] I want to be independent, I have to learn. They encouraged me to try different things like the computer, telephone, Internet. Even if I did not believe in my skills, they supported me” [PLF26].

“I am blind since birth, but I have always been struggling not to define myself through my disabilities. I am good at so many things. I love to discover new things. I hope I will always have the opportunity for learning” [PLM35].

The respondents often underlined that they have a high level of digital skills, which they are still improving. Many of them used smartphones, laptops, computers, braille notepads, white canes, and many professional applications [PLM31].

“I am curious about new technologies. I know these could replace my eyes. […] You can use words like ‘see’, etc. I am really able to conceptualize these words. I do not have problems with that. I am interested in new technologies and I use many of them every day. My digital skills are really high. […] I am working on digitalization of learning materials for people who are blind or vision impaired at university. I use my apps and professional computer programs. I wish to learn more. I would love to buy many of these devices, but they are too expensive for me. Finances limit many possibilities” [PLM34].

New technologies led to associations with the rights for privacy and independence. The access to digital devices improved blind adults’ well-being.

“I want to be independent and have privacy that I would not need to ask anyone for help. I want to learn new digital skills, to try with digital devices I have never had the opportunity to work with. I know that possibilities are great, but sometimes simple barriers stop us from self-development. I think financial barriers are the most common in Poland” [PLF37].

The interviewees wished to learn more about technological development and its possibilities for improving their lives. These attitudes of curiosity and being open-minded reflected positive experiences from contact with such devices.

“I wish to learn how to use assistive robots. First of all, I wish to have an assistive robot (laugh). Not all of the blind people I know are so optimistic about new technologies. Many depend on their parents or caregivers. If they do not know how to use computers, they do not understand why it is so important for us. […] These solutions are too expensive. I don’t know anyone who can afford these without support from PFRON (authors’ note: State Fund for the Rehabilitation of the Disabled)” [PLF26].

“I want to learn more about computer programs for blind adults. Now, I prefer to use my self-phone instead of my computer. But I know that there are so many new solutions for blind people worldwide. I know that many of them are out of my reach. I can’t afford them but I wish to have them” [PLM22].

### 4.2. Having to Learn—“If You Do Not Have New Digital Devices or Do Not Know How to Use Them, You End Up on the Margins of Society”

Learning new digital skills evoked many associations among the respondents with the feeling of a specific necessity of fitting to the changing situation. They mentioned the notions of feeling the pressure of the time and being on the margins of society.

“Sometimes, I feel the pressure of time. Technology gets ahead of me and I am left behind. I do not want to be isolated from society, so I have to learn new skills, usage of new devices. There is no other option for me. We live in a digital society. I know I really have to learn, instead of all difficulties” [PLM46].

“I am not so good at computers, smartphones. I use a white cane, I cannot imagine my life without that. I have to learn more about my smartphone functions. My colleagues who are vision impaired use many more functions of smartphones than I do. They told me how helpful these might be, even in buses or in moving around the city” [PLF39].

The interviewees were aware of the social and economic context of technological development. It implies the necessity of keeping pace with new technologies.

“I have to learn because I am not as good as I want to be in this respect. I know I can do more and I have to learn because time is passing by very fast. If they learn how to use these programs, devices, it will be easier for me in the future” [PLM38].

“I do not know if I want to learn, but I know I have to learn (laugh)” [PLF27].

“I have this feeling of necessity. I myself experience on a daily basis how helpful even simple smartphone apps are” [PLF45].

The respondents mentioned the role of the family, friends, and caregivers in supporting and motivating blind people for improving digital skills and experimenting with new digital solutions.

“It is difficult. I know I have to learn more about new technologies for blind people, but it is not so easy. It is easy to say, but without help from others, it would be impossible for me” [PLM42].

### 4.3. Being Socially Excluded—“Designers of New Technologies Do Not Think About Blind Adults”

The respondents wanted to take part in the program of designing new technologies for blind people. They saw many possibilities of improving their life through simple modifications of digital devices and applications.

“I was looking for a job in the learned profession of translation, but unfortunately I didn’t get one. The employer required the use of a certain programme that was not available to me. Often the problem is that various devices are not synchronized with programmes for the blind. I needed a workstation with a computer with a speaking programme” [PLM33].

“For designers of new technologies, blind people are a niche target group. At least in Poland this is the case. Many of these devices are so expensive that no one can afford to buy them. External support is needed, for example from the Fund for Assistance to People with Disabilities. These barriers are insurmountable” [PLF24].

The respondents perceived blind people as well educated about new technologies and having positive attitudes toward these kinds of solutions. They focused their attention on the problems with finding jobs for blind adults because of some technical aspects of non-synchronized computer equipment with programs and applications for the blind.

“I’ve been blind since birth and they’re really great at operating different devices. I even think I do better than people who lost their sight later. They are more ashamed of their disabilities and have problems asking for help. I have no such inhibitions. New technologies are my natural environment, but I still feel excluded. Many workplaces don’t have computers or workstations in general that are adapted to the needs of blind people” [PLF25].

“It is known that every person has different needs, but it seems to me that some devices could be combined somehow. So that when developing some technologies, for example for deaf people, we do not neglect the needs of others. We could immediately think about how to connect different categories of people using the same technologies. I have the impression that we are often divided by access to new technologies, yet they have the potential to connect people. Designers of new technologies should be more focused on this” [PLF37].

The respondents highlighted the problem of incompatibility between different digital devices. They felt that these technical problems are not so complex that they cannot be fixed by the designers of these devices.

“There are many different devices on the market. I have read about it, but unfortunately they are not available to us. The barrier is usually money. It would be much easier to exploit the potential of cheaper digital devices like a computer, laptop or smartphone. Most of the software used in office work is not synchronised in, for example, the speech synthesiser on the phone” [PLM46].

“In the digital world, it is the same as in the human world. The problem is communication. Many devices do not work with free apps for blind people. I have not heard of any foundations developing free software for blind people. The kind along the lines of search engines or text programs. We’re too small a group, it’s probably not worth it to anyone” [PLM38].

“I would love to take part in such an experiment where they test some new programme or device. I’ve had really different experiences in my life. On more than one occasion, while moving around the city, I came across information signs written in Braille…only that the lettering was upside down” [PLF45].

The respondents also highlighted problems for blind people that are related to where they live. Poland was perceived by them as a less friendly country in this respect. The study was convinced that, in countries that are more economically developed, the situation of blind people is definitely better, thanks, among other things, to access to the latest digital innovations for the blind.

“In Poland, we use simple and cheap devices. We do not have access to the more expensive ones. Even at the various workshops for us, the workshops are not equipped with more modern equipment. It seems to me that in more developed countries, blind people are not so socially isolated, it is easier for them to find work” [PLF39].

The respondents were interested in cooperation with designers of new technologies. They pointed to the social potential of such experiments.

“I have been waiting for such an invitation for a long time. There is so much I could offer. I have a lot of knowledge about new technologies and a completely different perspective. I have been blind since birth and would like to do something for the benefit of people like me. Could give advice on how to make different devices, programmes, applications more suitable for our needs. It would even be important to focus on the simplest devices that everyone has access to and synchronise them” [PLM31].

“If someone doesn’t take an interest in how to combine all these devices designed for the blind and the sighted, then I really will never find a job compatible with my education. I feel socially excluded because of such problems. I really want to work among other people” [PLM35].

### 4.4. Fearing to Stop Understanding—“Technological Development Is Too Rapid, It Is Difficult to Be on Time While Ageing”

The respondents were concerned that they might not be able to keep up with the development of new technologies in the future. In their experience, change is very rapid even today.

“I happen to think about ageing. What I fear most is digital exclusion. […] Sometimes, I am afraid that I would be alone, that I would be afraid to go out, because I would not know how to move in the city. If I stop keeping up with these innovations, I’ll end up on the margins of society. Already today, not being able to use a computer, or a Braille ruler integrated into a computer, means that we start to be out of touch with others. Without a smartphone, there is no point in going out on the town. And what will happen in the future? All this will intensify even further” [PLM42].

“The world is changing so quickly. I’m afraid I won’t be able to keep up with technological advances, that I won’t know how to use the new smartphone models and I’ll be afraid to leave the house” [PLM38].

“What scares me is that one day he won’t have anyone to help him with his laptop, or maybe even a robot” [PLF39].

“It is difficult to talk about the future because the present is changing very quickly. This is almost evident in the new technologies. Something new is emerging all the time. Of course, I don’t have access to everything. I’m afraid I won’t be able to keep up with it all” [PLF37].

The respondents linked not keeping up with new technologies to social isolation, exclusion from society, lack of access to modern services, unemployment, etc.

“I’m afraid of such a stagnant life that I’ll be locked up at home, unable to meet other people by not being able to keep up with all the news. […] Now I’m supported by family and friends. And what will happen in the future?” [PLF45].

When I think about it, what I fear most is the loss of independence. […] I also fear that I won’t be able to cope with new laptops or smartphone apps in the future. After all, it’s all changing so quickly” [PLF39].

The respondents had rather good experiences with learning to use new models of mobile phones or computers. However, these positive experiences did not translate into a more positive assessment of an uncertain future.

“I remember how smartphones displaced such simple analogue phones. At the time, I was worried that I wouldn’t know how to use them. I learned quickly, but as I think about the future, how many new things are being developed, it fills me with a bit of anxiety. Will I ever be able to cope with it all? Technology is developing too fast” [PLM46].

### 4.5. Waiting for the Changes—“I Hope My Situation Will Change in the Future”

The respondents expressed hope that the development of new technologies in the future could have a breakthrough impact on the treatment of blind people and the use of new technologies in improving their various competences. New technologies were often associated with improving the quality of life of blind people.

“I am not afraid of ageing or new technologies. I hope to change my life for the better with them in the future” [PLM34].

“There used to be no known cure for many diseases that are successfully treated today. No one dreamt that robots would perform medical operations. So I hope that the development of new technologies will help me in the future. I believe in it deeply” [PLF24].

The interviewees also expressed the hope that, in the future, designers of new technologies will be more sensitive to the needs of older people.

“I wish there were more age-friendly technologies. […] One that makes life easier for older people. As a blind person, I would dream of such a special coffee machine, for example, or a cleaning robot. I would like to have an assistive robot to help me get around town” [PLF27].

“Age-friendly technologies are certainly ones that will support us greatly on a daily basis. They will help us in our daily duties. But not only, because the most important thing will be such social support when the help of another person is missing. I would like to have such a robot that reminds me of things and speaks back to me. Sometimes I feel lonely” [PLM33].

Respondents tended to mention older technological solutions for blind people in their statements, such as a speech synthesizer, a white cane, a Braille keyboard, or free apps on a smartphone. Some respondents mentioned assistive robots as examples of modern solutions. The interviews did not talk about the latest technologies for blind people available on the market.

“I am curious to see what the Braille notepad will be replaced with in the future. I am also curious to see how the various geolocation services for blind people will develop. I hope that the villages, towns and cities of the future will be really smart. New technologies need to be more compatible with us. I look forward to that” [PLF26].

## 5. Discussion

As there is a limited amount of research that incorporates blind adults’ experiences of adapting to new technologies, as well as their expectations of the designers of these kind of solutions, this study set out to explore these issues. This grounded theory study is framed as a specific methodological investigation that can lead to further complementary formulation of the factual theory. The second assumption is that it can also lead to the discovery and elaboration of gaps in the formal theory. To achieve these goals, the next step was to bridge the gap between the immediacy of the factual theory and the abstract generality of the chosen formal theory, as characterized in the literature review section. We already know the results of the research, but the question of using a valid theoretical framework for analyzing these findings is still open. What do our findings contribute to the state of sociological science? To what extent do they fit into the established discourses, approaches and theories? Based on them, can we attempt to forge a new research path or bring concepts that have been somewhat forgotten by sociologists back into the mainstream of analysis?

A comparison of the research results to previous findings leads to interesting conclusions. We discuss these conclusions by referring to the description of selected works from the literature in Section 2 of the paper. The previous studies have directed attention toward the need to create blind-friendly spaces [51]. This research has mostly been conducted, among other things, within the theoretical framework of the smart-city concept [52,53]. The results of our interviews show that blind adults have a good perception of such solutions, but often, in their opinion, they are not fully adapted to the needs of blind and/or visually impaired people. These people have never been involved in the design process of such solutions. However, they themselves claim that many of these facilities are inadequate to meet their needs, e.g., information boards written in Braille with errors. The smart city approach therefore appears to be an interesting analytical perspective for describing the adaptation process of blind people to new solutions based on technological developments. In this research, the development of the technology itself comes to the fore. The issues of individual service recipients, primarily people with disabilities, seem to be placed in the background of the analysis. It is assumed that these people will adapt to the proposed solutions, which are not always designed in collaboration with the social actors involved. This topic seems to be particularly important in developing countries or countries where blind people are identified as a category of people at risk of social exclusion due to specific cultural and social conditions.

Our respondents often experienced being unnoticed by the developers of new technologies. They described themselves as an unimportant category of consumers of such solutions. In their statements, they also often pointed out the lack of interest in their problems on the part of free software developers. Most of the respondents were able to list the main problems related to their everyday functioning in a technologized environment. The biggest problems in finding a job were the unsuitability of workstations for blind people. In addition, many applications, programs, and software usually used at work are not compatible with programs designed for the blind. Our respondents often talked about their experiences in looking for a job and the barriers, simple in their opinion, resulting from the incompatibility of different computer programs. They also claimed that they would be able to offer a simple technological solution to such problems in a specific workplace, provided that the employers expressed interest. The interview participants expressed the belief that employers do not want to work with blind people because negative stereotypes dominate their thinking. Furthermore, they are often afraid to employ blind people because they have little knowledge of the potential of blind people. The design of age-friendly technologies for blind people may have addressed these very issues. It can be assumed that blind people living in developing countries and/or experiencing financial difficulties are affected by these problems. In the literature, most attention is given to various advanced technological solutions [62,63]. A wide variety of research has studied how technology can assist visually impaired people [64]. The most common theme of the papers on the development of assistive technologies for blind people is the development of various digital solutions, such as assistive technologies, learning technologies and artificial intelligence, etc. It should be emphasized here that there are many such digital devices, which are often very technologically advanced and which are designed for blind people. We are witnessing constant technological advances, and therefore, more technologically advanced devices. Importantly, they are not easily accessible to blind people in many countries. The most common barriers to accessibility are financial and educational ones [65]. A much-overlooked issue is the compatibility of older technological solutions with existing solutions. In our opinion, this is a very important issue because, as research shows, as people get older, they are less willing to test various digital innovations. Rapid technological development usually leads to old devices being replaced by new ones. This can lead to many negative consequences for categories of people at risk of digital and social exclusion. When we are talking about new technologies, the participants in our interviews first mentioned devices that have been on the market for a long time, such as Braille rulers, speech synthesizers, geolocalizers or smartphones.

The previous findings suggest that blind people are perceived as having low levels of digital competence [66]. Furthermore, in the literature we find claims that family members and/or carers have the greatest influence in shaping blind people’s attitudes toward digital technologies [67]. Our research did not confirm these findings. It should be considered that the level of digital competence of the respondents was good. They all used computers, smartphones, and many applications for blind people on a daily basis. They also had a good knowledge of commercially available digital devices, robots, learning technologies, and other applications for blind people. Economic and educational reasons were the main causes for not using digital devices and/or assistive technologies, as cited by the interviewees.

Importantly, it can be deduced from the experiences of the participants in the interviews that the attitudes of those closest to them toward new technologies were, in some cases, an important factor in motivating blind people to improve their digital skills. Of much greater importance was the way blind people’s families and/or carers perceived their social and professional roles. On the basis of our research, it can be concluded that stereotypes existing in specific societies should be seen as the main mediating factors in the process of blind people entering the labor market. Our study participants had precise dreams and expectations with regard to work, while the majority of them did not perform work that was in line with their education.

The main thread of the research was the issue of designing new age-friendly technologies for blind people [68,69]. The results of the research were surprising to us, as our interviewees did not mention the names of specific technologies, their functionality, appearance, etc., in their statements. The question about age-friendly technologies for the blind evoked associations with the process of keeping pace or not keeping pace with technological developments. Age-friendly technologies are those that are accessible and easy to use. Most of the respondents had concerns about their future, as they were aware that their social activities were already linked to their ability to use various digital devices. The respondents did not perceive ageing as a race against time, but far more often as a race against the development of digital technologies.

In conclusion, our research demonstrates the importance of including blind people in the design process of age-friendly technologies. These people, thanks to their daily experiences with different technologies, can help to develop solutions adapted to the needs of this category of people. Designers of new technologies may overlook the existence of some technical problems related to the incompatibility of different software, and it may be very easy to solve them. Moreover, such facilities may also be more accessible to blind people who are experiencing financial difficulties.

An important topic for sociological research in this area should therefore be a survey of the attitudes among a representative sample of employers in Poland. A second emerging research perspective is to conduct qualitative research among new technology designers in Poland, especially within start-ups emerging in the environment of university centers. The main issue of the research would be how blind people are perceived by designers of the age-friendly technologies as potential co-creators, testers and users of the new solutions.

Finally, we would like to refer to the theories within which the problems of blind people are described. Theories and concepts of social inclusion seem to pay too little attention to the importance of assistive technologies in the process of blind people’s entry into societies and their active ageing [70,71,72]. In our opinion, these issues should also be analyzed within the concept of the network society or digital society. Participants in the interviews perceived themselves as living in a high-tech society and dependent on access to such solutions during their daily activities. It seems that within this conceptual framework, it would be particularly interesting to analyze the processes of inclusion and social integration of blind people. This approach seems all the more valid as we find descriptions of studies in the literature on the social activity of blind people. The results of our research will therefore lead to new research questions. We see the clear need to conduct more studies within the indicated methodological, problematic and theoretic frameworks to find the answer to these questions. The adaptation of blind people to new technologies is a very complex issue. Many factors mediating this process are culturally based ones. The questions we asked to find out about blind people’s experiences and expectations of using specific devices and the expectations of the designers of new technologies have brought to mind the need to keep pace with technologies while we are ageing. This is a very interesting conclusion, which directs our attention towards the need to further develop theoretical frameworks that facilitate the understanding of these processes and give the hope of finding solutions to the problems for blind people.

## 6. Research Limitations and Directions for Future Research

The findings display some limitations due to the research method applied. The qualitative research does not provide data enabling a holistic and systemic approach to the problem. However, the chosen method seems to be the most appropriate for identifying blind adults’ experiences of adapting to new technologies. The most appropriate approaches to its research are those associated with the humanistic stream, i.e., ones in which emphasis is placed on understanding the way blind adults experience and perceive technological development. A microlevel approach seems to be the most appropriate in that context, despite it being difficult to make general comparisons. In the future, we plan to conduct a focus-group study among the blind adults. The second avenue for future research is a survey of the attitudes among a representative sample of employers in Poland. The third one is to conduct qualitative research among new technology designers in Poland.

## 7. Conclusions

A grounded theory study on blind adults’ experiences of adapting to new technologies was focused on how they have been experiencing changes implied by digital transformation, how they assess the possibilities of improving some already existing solutions, and what specific needs and expectations they regarding the design of such solutions. The next undertaken issue was the assessment of the potential for self-development by using digital technologies and improving individuals’ digital skills. Sixteen blind adults took part in the in-depth interviews. The data analysis revealed five distinct categories that captured these experiences and expectations: (1) wishing to learn—“Digital technology gives me privacy and independence”; (2) having to learn—“If you do not have new digital devices or do not know how to use them, you end up on the margins of society”; (3) being socially excluded—“Designers of new technologies do not think about blind adults”; (4) fearing to stop understanding—“Technological development is too rapid, it is difficult to be on time while ageing”; and (5) waiting for the changes—“I hope my situation will change in the future”. Together, these five categories form the basis of the core category “Ageing and keeping pace with technology”, which encapsulates the initial adaptation experiences of the interviewees to the technological development. The findings indicate that the blind adults experienced digital devices as tools for improving their well-being, but they also viewed them as posing the threat of being socially excluded because of technology designs and accessibility barriers. Aging evoked associations with the issue of keeping pace with technology. There is a need to develop an effective social environment for the cooperation between blind adults and the designers of new technologies. The analysis of the transcripts focused on the need for the implementation of specialized training to improve digital competences for the blind and the development of systemic programs to support the development of blind-friendly workplaces in Poland. A third important issue is the interest in blind people’s problems resulting from the incompatibility of many programs with blind solutions by free-software developers and the foundations funding such initiatives.

## Figures and Tables

**Table 1:** Sociodemographic characteristic of respondents.

Code *	Gender	Age
PLF26	Female	26
PLF24	Female	24
PLM31	Male	31
PLM35	Male	35
PLF37	Female	37
PLM42	Male	42
PLM22	Male	22
PLM34	Male	34
PLF25	Female	25
PLF45	Female	45
PLF26	Female	26
PLF39	Female	39
PLM46	Male	46
PLM33	Male	33
PLF27	Female	27
PLM38	Male	38

* Nationality, gender, and age.

## Data Availability

The data supporting the reported results can be found at the Institute of Sociological Sciences of The John Paul II Catholic University of Lublin. Availability is upon researcher’s request, according to the politics of data management at The John Paul II Catholic University of Lublin: alina.betlej@kul.pl.
